# Impact of Heart Rate Monitoring Using Dry-Electrode ECG Immediately After Birth on Time to Start Ventilation: A Randomized Trial

**DOI:** 10.3390/children12081082

**Published:** 2025-08-18

**Authors:** Siren Rettedal, Amalie Kibsgaard, Frederikke Buskov, Joar Eilevstjønn, Vilde Kolstad, Jan Terje Kvaløy, Peder Aleksander Bjorland, Hanne Pike, Joanna Haynes, Thomas Bailey Tysland, Peter G. Davis, Hege Ersdal

**Affiliations:** 1 Department of Simulation-Based Learning, Stavanger University Hospital, 4068 Stavanger, Norwayvilde.34c@live.no (V.K.);; 2 Faculty of Health Sciences, University of Stavanger, 4036 Stavanger, Norway; 3 Strategic Research, Laerdal Medical, 4002 Stavanger, Norway; 4 Department of Research, Section of Biostatistics, Stavanger University Hospital, 4068 Stavanger, Norway; jan.t.kvaloy@uis.no; 5 Department of Mathematics and Physics, University of Stavanger, 4036 Stavanger, Norway; 6 Department of Pediatrics, Stavanger University Hospital, 4068 Stavanger, Norway; 7 Department of Clinical Science, University of Bergen, 5021 Bergen, Norway; 8 Department of Neurology, Stavanger University Hospital, 4068 Stavanger, Norway; 9 The Royal Women’s Hospital, University of Melbourne, Parkville, Melbourne, VIC 3052, Australia

**Keywords:** dry-electrode ECG, heart rate, heart rate monitoring, newborn resuscitation, positive pressure ventilation, newborn resuscitation algorithm

## Abstract

**Highlights:**

**What are the main findings?**

- Ventilation was provided within 60 s to 45% of newborns in the ECG heart rate-visible group versus 33% in the control group, *p* = 0.12. Time from birth to start of PPV was a median of 66 (44, 102) s in the intervention and 84 (49, 148) s in the control group, *p* = 0.058.
- Only 36% of resuscitated newborns were bradycardic with HR < 100 beats per minute at the start of PPV.

**What is the implication of the main finding?**

- Use of dry-electrode ECG HR monitoring did not change the proportion of newborns that received PPV within 60 s after birth, but early termination due to employee protest over video recording rendered the trial inadequately powered to detect a difference. Starting ventilation within 60 s after birth remains challenging.
- All newborns that received PPV were apneic or ineffectively breathing at the start of PPV, but only about a third were bradycardic. This reinforces current resuscitation guideline recommendations, highlighting that respiratory status is the most important indicator for starting PPV.

**Abstract:**

Background/Objectives: Newborn heart rate is an integral part of resuscitation algorithms, but the impact of ECG monitoring on resuscitative interventions and clinical outcomes has been identified as a knowledge gap. The objective was to evaluate the impact of routine use of dry-electrode ECG in all newborns immediately after birth on time to start positive pressure ventilation (PPV) when indicated. Methods: We conducted a randomized clinical trial from June 2019 to November 2021 at Stavanger University Hospital, Norway. Dry-electrode ECG sensors were applied immediately after birth to all newborns ≥ 34 weeks’ gestation. Randomization determined whether the heart rate display was visible or masked. Time of birth was registered in an observation app. Time to start ventilation was calculated from video recordings. Results: In total, 7343 newborns ≥ 34 weeks’ gestation were enrolled, 4284 in the intervention and 3059 in the control group, and 3.7% and 3.8% received ventilation, respectively. In 171/275 (62%) of the newborns the exact time of birth and a video of the resuscitation were available, for 98 in the intervention and 73 in the control group. Ventilation was provided within 60 s to 44/98 (45%) in the intervention and 24/73 (33%) in the control group, *p* = 0.12. Time from birth to start of PPV was a median of 66 (44, 102) s in the intervention and 84 (49, 148) s in the control group, *p* = 0.058. Resuscitated newborns were apneic (74%) or breathing ineffectively (26%) at the start of PPV, and only 36% had a heart rate < 100 beats per minute. Conclusions: The use of dry-electrode ECG heart rate monitoring did not change the proportion of newborns that received ventilation within 60 s after birth, but early termination due to employee protests to video recordings rendered the trial inadequately powered to detect a difference. Breathing status was likely a more important determinant of starting ventilation than bradycardia.

## 1. Introduction

At birth, heart rate (HR) is used to assess the effectiveness of spontaneous breathing and the need for positive pressure ventilation (PPV) and as a marker of response to resuscitation [1]. Therefore, a rapid and reliable method of measuring HR is considered a critical adjunct for neonatal resuscitation [1]. The International Liaison Committee on Resuscitation (ILCOR) suggests using electrocardiogram (ECG) for rapid and accurate estimation of HR in newborns requiring resuscitation, when resources permit [2,3]. According to ILCOR recommendations and European and American resuscitation guidelines PPV should commence within 60 s of birth if the newborn fails to establish spontaneous and effective breathing following assessment and stimulation, and/or the HR is persistently bradycardic < 100 beats per minute (bpm) and/or HR decreases if initially satisfactory [1,4,5]. Compliance with these recommendations is challenging. There is considerable variation in the time from birth to the start of ventilation and often an important delay in commencing this lifesaving treatment [6,7,8].

Although newborn HR is an integral part of resuscitation algorithms, the impact of the routine use of ECG monitoring immediately after birth on resuscitative interventions and clinical outcomes has been identified as a knowledge gap by ILCOR [1,2]. Limitations in existing technology have made it difficult to obtain rapid, accurate, and continuous HR measurements during the first minutes of life, when important decisions are made on resuscitative actions [9,10,11]. NeoBeat (Laerdal Medical, Stavanger, Norway) is a reusable, wireless dry-electrode ECG device developed to provide continuous measurement of HR after birth and during resuscitation. The device can be applied around the newborn’s thorax or upper abdomen within 3–6 s after birth without drying of the skin [9,12] and a reliable HR is digitally displayed within 3–13 s of application [9,12,13,14]. The HR obtained by dry-electrode ECG correlates well with that of conventional gel-electrode ECG [9,14,15].

In this randomized controlled trial (RCT), we evaluated the effect of routine HR monitoring by dry-electrode ECG on all newborns immediately after birth on resuscitation practices. The main outcome was the proportion of resuscitated newborns who received PPV within 60 s after birth, in line with resuscitation guidelines.

## 2. Materials and Methods

### 2.1. Setting

The trial was conducted at Stavanger University Hospital, Norway, the region’s only hospital providing tertiary-level obstetric and neonatal services, for a population of 350,000 with around 4300 annual deliveries. Delayed cord clamping (≥60 s) is performed for vigorous newborns, while those in need of resuscitation have the cord cut immediately and are taken to a resuscitation table in a separate room (mean distance 12 m). Cord milking is not routinely practiced.

### 2.2. Study Design

This was a single-center, two-armed, parallel RCT conducted from 6 June 2019 to 16 November 2021 [16]. A dry-electrode ECG was applied to all eligible newborns immediately after birth. Randomization determined whether the HR display was visible or masked. Computer-generated block-randomization with variable block sizes was used, and reallocation of study groups was performed by a research assistant on a weekly basis during the study period (quasi-randomization). The intervention could not be blinded for the trial participants or healthcare providers (HCPs), but outcome assessors were blinded to the intervention.

This study was approved by the Norwegian National Research Ethics Committee West (2018/338, dated 27.04.18) and registered in Clinicaltrials.gov (NCT03849781).

### 2.3. Study Participants

Expectant parents were identified and asked to participate during the routine ultrasound screening visit early in pregnancy. In addition, those not already contacted were approached on admission to the labor ward. In-born newborns at ≥34 gestational weeks were eligible for inclusion. In the final analysis, we included newborns who received PPV within 5 min of birth, with the exact time of birth registered and the resuscitation captured on video. Newborns were excluded if born with congenital malformations interfering with the placement of the dry-electrode ECG device, such as gastroschisis. Employees could decline participation by requesting the deletion of video recordings of resuscitations.

### 2.4. Intervention

**Figure 1:**
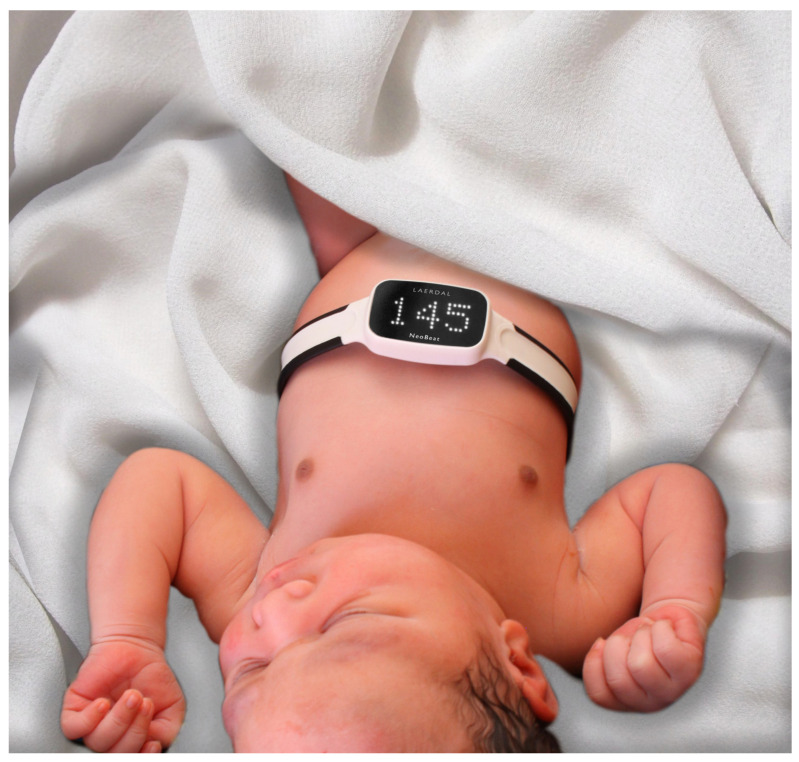
Image of NeoBeat placed on newborn (Copyright permission obtained).

The dry-electrode ECG device NeoBeat (Laerdal Medical, Stavanger, Norway) was placed on the abdomen or chest of the participating newborns by the midwife or nurse assistant immediately after vaginal births, as shown in Figure 1. For caesarean sections, the device was placed when the newborn was clear of the operating field or on the resuscitation table. For the newborns assigned to the intervention group, the display was visible to guide decision-making and management. For the newborns assigned to the control group, the dry-electrode ECG device was placed to capture and store HR data with the display masked, and HR was not disclosed to the HCPs. Additional HR assessment could be performed at the HCPs’ discretion, either by palpation, auscultation, conventional ECG, and/or pulse oximetry. If the newborn was thought to need resuscitation, it was brought to the resuscitation table with the wireless dry-electrode ECG device in place. For newborns receiving chest compressions, the device was repositioned to the abdomen as necessary. The HCPs were asked to comply with the national resuscitation guidelines, stating that apnea or ineffective breathing or sustained HR < 100 bpm were indications for starting PPV within 60 s of birth.

### 2.5. Data Collection

Time of birth was registered in real time by the nurse assistant in the Liveborn observation app (Laerdal Global Health Stavanger, Norway). HR from the dry-electrode ECG device was transmitted via Bluetooth to the Liveborn observation app. The Laerdal Newborn Resuscitation Monitor (Laerdal Global Health) recorded and stored ventilation data, including applied peak inflation pressures (PIPs), positive end-expiratory pressures (PEEPs), tidal volumes, mask leak, and ventilation rates. Visual feedback on ventilation parameters was not displayed to the HCPs. Resuscitations were video recorded using motion sensor-triggered cameras placed above the resuscitation station, capturing the newborns and the hands of the HCPs. The timestamps in the video server and Liveborn observation app were automatically synchronized. The video recordings were reviewed, and the data were annotated by investigators (SR, AK, VK, HP) using XProtect Smart Client software V.2016 (Milestone, Copenhagen, Denmark) and ELAN 5.8 (The Language Archive, Nijmegen, The Netherlands). The video recordings were used to evaluate breathing effort at initiation of PPV, timing and duration of PPV, intubation, and chest compressions. Any inflation was defined as resuscitation with PPV. Information from patient records was electronically extracted and stored in the research database.

### 2.6. Outcome Measures

The primary outcome was the proportion of resuscitated newborns receiving PPV within 60 s of birth. Additional outcomes included time from birth to start of PPV. Safety outcomes included the proportion of live births receiving PPV in the two groups; PPV provided to newborns breathing spontaneously or PPV not provided to newborns not spontaneously breathing, as determined by video recordings; and, finally, damage to skin or organs by placement of HR sensor devices.

### 2.7. Statistical Analysis and Power Calculation

The sample size was calculated based on an earlier study from our hospital, where 35% of resuscitated newborns at gestational age ≥ 34 weeks received PPV within 60 s after birth [17]. We calculated that 169 newborns were needed in each group to detect an improvement of 50% (increase to 52.5%) receiving PPV within 60 s of birth, with a 5% overall significance level, 90% power, and one interim analysis midway [18]. Intention-to-treat analyses were performed for primary and secondary outcomes.

Demographics, resuscitation characteristics, and short-term clinical outcomes were compared between the intervention (HR-displayed) and the control (HR-blinded) groups. Background characteristics were summarized as means/standard deviations or medians/quartiles for continuous variables, according to their distribution, and as frequencies and percentages for categorical variables.

The Wilcoxon rank sum test was used to test for differences between groups for continuous variables, and Fisher’s exact test was used for categorical variables. Cumulative distribution functions for time to PPV were estimated by the Kaplan–Meier estimator. HR data processing, data point extraction, and statistical analysis were performed using MATLAB R2024a (MathWorks, Natick, MA, USA) and R V.4.3.2 (R Core Team).

### 2.8. Early Termination of the Trial

No HCPs requested deletion of their resuscitation videos in the period from 6 June 2019 to 16 November 2021. However, the study was temporarily halted on 17 November 2021 due to employee protests over the video recording of resuscitations. Data collection resumed on 4 November 2022 with a QR code for anonymous deletion of videos. A surge in deletion requests after 4 November 2022 compromised the trial’s conduct and generalizability, leading to early termination without reaching the final sample size. We report the data from approximately half of the planned sample size, collected from 6 June 2019 to 16 November 2021.

## 3. Results

### 3.1. Baseline Characteristics

**Figure 2:**
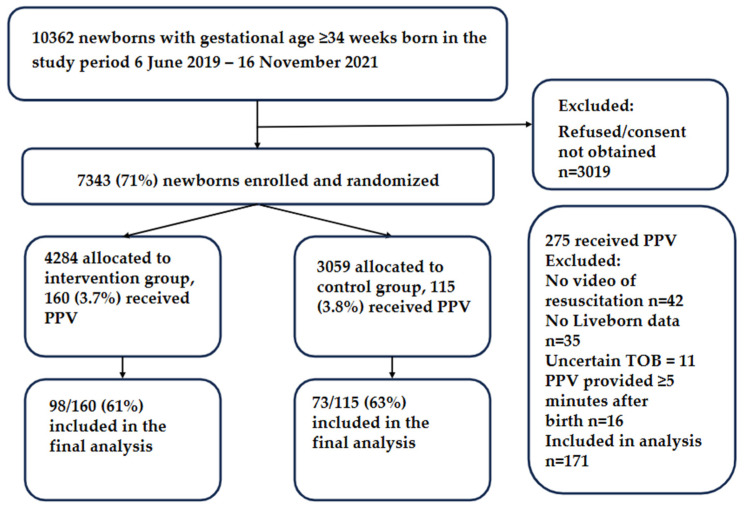
Flowchart of the study participants.

In total, 10,362 newborns with gestational age ≥ 34 weeks were born during the study period, of whom 7343 (70.9%) were enrolled in the study: 4284 in the intervention group and 3059 in the control group. Among the newborns who received PPV within 5 min of birth, the exact time of birth and videos of resuscitations were available for 98/160 (61%) in the intervention and 73/115 (63%) in the control group. A flowchart of the study participants is shown in Figure 2.

There were no important differences in the maternal and newborn characteristics between the groups among the resuscitated newborns (Table 1).

### 3.2. Resuscitative Interventions and Short-Term Outcomes

**Figure 3:**
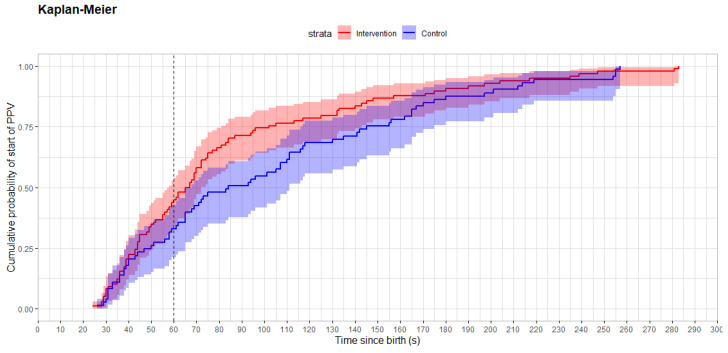
Kaplan–Meier plot showing time from birth to start of PPV for intervention (red) and control (blue) participants.

The proportion of resuscitated newborns who received PPV within 60 s after birth was 44/98 (45%) in the intervention group and 24/73 (33%) in the control group, *p* = 0.12. Time from birth to start of PPV was median 66 (44, 102) s in the intervention group and 84 (49, 148) s in the control group, *p* = 0.058. The resuscitation characteristics and short-term clinical outcomes are presented in Table 2 and Figure 3.

PPV was indicated in all resuscitated newborns as evaluated by video, as 122 (74%) were apneic and 43 (26%) were breathing ineffectively at the start of PPV (data available for 165 newborns).

Time from birth to arrival at the resuscitation table was similar in both groups: the median (quartiles) was 40 (28, 62) s in the intervention (data available for 91 newborns) and 46 (31, 71) s in the control group (data available for 70 newborns), *p* = 0.3.

No differences in the ventilation parameters were detected between the groups (Table 2).

### 3.3. Heart Rate Acquisition

Among all the enrolled newborns, 5465 had HR captured within the first 10 min after birth. Time from birth to first dry-electrode ECG skin contact was median (quartiles) 5 (3, 10) s. Time from birth to first HR displayed on the dry-electrode ECG device was 16 (10, 30) s, and time from first dry-electrode ECG skin contact to first recorded HR was 7 (4, 13) s. The first measured HR was 152 (115, 174) bpm.

Among newborns receiving PPV, HR at 60 s was 147 (88, 167) in the intervention group (data available for 56/98 newborns) and 139 (102, 168) in the control group (data available for 44/73 newborns). HR at start of PPV was 146 (86, 177) and 106 (75, 157), *p* = 0.065, respectively (data available for 49/98 and 44/73 newborns).

Dry-electrode ECG HR was available prior to PPV in 59/98 (60%) of the newborns in the intervention group, at a median of 19 (13, 46) s. In 29 intervention cases, dry-electrode ECG HR was first registered after the start of PPV, and in 10 cases, dry-electrode ECG HR was not collected. Although not displayed, HR was registered by the dry-electrode ECG prior to PPV in 50/73 (68%) of the newborns in the control group, at a median of 28 (15, 48) s.

The proportion of all newborns with at least 10 s of collected HR < 100 bpm within the first 5 min after birth was 13.5% (479/3551) in the intervention group and 15.9% (392/2466) in the control group, *p* = 0.01. Among the resuscitated newborns, the proportion of newborns with at least 10 s of recorded HR < 100 bpm within the first 5 min after birth was 43% (37/86) in the intervention group and 60% (35/58) in the control group, *p* = 0.06. When analyzed for newborns bradycardic immediately after birth, time to HR ≥ 100 bpm was similar between the groups, at a median of 64 (35, 95) (n = 29) vs. 59 (41, 77) (n = 17) seconds, *p* = 0.64.

### 3.4. Safety Outcomes

There was no difference in the proportion of newborns who received PPV between the intervention and control groups: 160/4284 (3.7%) and 115/3059 (3.8%), respectively. In the resuscitated newborns with video recordings, all the newborns were either not breathing or breathing ineffectively at the start of PPV. There was no reported difficulty with the application of the dry-electrode ECG sensors. Furthermore, there were no cases of damage to skin or organs reported due to the dry-electrode ECG device, gel-electrode ECG, and/or pulse oximetry.

### 3.5. Protocol Violations

Protocol violation was observed in the video recordings of six participants in the control group, where the HCPs removed the tape masking the dry-electrode ECG display when the HR could not be obtained reliably by standard HR assessment during resuscitation. Unmasking of the display occurred at a mean (range) of 101 (1–206) seconds after initiation of PPV and did not affect the primary and secondary outcome measures, proportion of newborns receiving PPV within 60 s after birth, or time from birth to start of PPV. In five cases (four intervention cases and one control case), the dry-electrode ECG device was not placed on the newborn as determined by the video of the resuscitation table and the HR data from the Liveborn observation app. All randomized participants were included in the analysis according to the intention-to-treat principle to minimize the risk of bias.

## 4. Discussion

This is the largest RCT evaluating the impact of dry-electrode ECG monitoring immediately after birth. The proportion of newborns who received resuscitation with PPV did not differ between the HR-visible and HR-masked groups. In total, 45% of the resuscitated intervention newborns and 33% of the resuscitated control newborns received PPV within 60 s after birth. The median time from birth to the start of PPV was 66 and 84 s, respectively. However, since this study was halted early and the results were not statistically significant, the outcome remains inconclusive.

It is a common perception that most ventilated newborns are bradycardic with HR < 100 bpm at the start of PPV. However, only 36% (33/92) of those who had HR recorded at the start of PPV were bradycardic at the start of PPV in this study. Indications for resuscitation were met in all newborns, as 3/4 were apneic and 1/4 were ineffectively breathing at the start of PPV. Breathing status was therefore a more important determinant of starting ventilation than bradycardia.

Newborn resuscitation algorithms reflect the principle that starting resuscitation in non-vigorous newborns is time critical [4]. Both delays in starting and interruptions in PPV may impair recovery [19,20]. Although not demonstrated in high-resource settings, a study in a low-resource setting found a 16% increase in the risk of death or morbidity for every 30 s delay in starting PPV among 459 newborns [19]. Another study demonstrated that for each 1 bpm increase in first detected HR after birth, the risk of death was reduced by 2%. A rapid increase in HR to >100 bpm in response to ventilation was associated with a 75% reduction in the risk of death. Conversely, a decrease in HR to <100 bpm when PPV was paused was associated with an almost twofold increased risk of death [20]. However, both obtaining reliable HR and initiating PPV within 60 s of birth have proven challenging in both high- and low-resource settings [7,8,21,22,23].

There is limited evidence on whether the routine use of ECG monitoring at birth and during neonatal resuscitation improves clinical outcomes [2,24]. Patterson et al. performed a pre–post clinical trial using dry-electrode ECG in non-breathing newborns in a low-resource setting. They found that time to bag mask ventilation decreased by 64.3 s during HR-guided resuscitation [25]. Despite continuous ECG HR monitoring, 20% of newborns classified as stillborn by the skilled birth attendants were actually liveborn [26]. Similar findings were reported by Ersdal et al., who concluded that distinguishing fresh stillbirth from severely asphyxiated newborns is clinically challenging [27]. A pre–post observational study by Shah et al., conducted in a high-resource setting enrolling 632 newborns receiving PPV at birth, showed no difference in mortality, but a lower proportion of intubated newborns and increased odds of receiving chest compressions when ECG monitoring was used [28]. Katheria et al. performed a pilot RCT, enrolling 40 preterm newborns with mean gestational age < 30 weeks, to determine whether displayed ECG-HR had an effect on clinical interventions during stabilization at birth. Although clinical interventions were started earlier in the HR-displayed group, the findings did not reach statistical significance [29]. Abbey et al. randomized 51 newborns with gestational age < 31 weeks to HR-displayed and HR-blinded groups; they found no difference in time to stabilization, defined as time from birth to achieve HR ≥ 100 bpm and oxygen saturation within goal range [30]. Another pilot RCT, including 42 newborns of <33 weeks’ gestation, compared a novel ECG algorithm displaying HR earlier compared to the conventional ECG algorithm. Newborns randomized to the novel ECG group had HR displayed faster, and PPV initiated sooner, compared with conventional ECG. Time for display of ECG HR from birth was 161 (56) versus 111 (28) s for the conventional ECG algorithm versus the new algorithm. In total 22 of the 42 participants required PPV, and HR was available prior to the start of PPV in only 2 (9%) of the 22 participants [23]. This is in contrast to the present study using dry-electrode ECG, where 60% of the newborns in the intervention group had HR available before the start of PPV, at a median of 19 s after birth. We previously demonstrated that dry-electrode ECG displays HR 85% of the time from birth through resuscitation with PPV, compared to 25% with standard ECG [9].

Neonatal resuscitation guidelines state that PPV should be started if the newborn is not breathing effectively, or if HR remains low after the initial steps, including tactile stimulation [4]. Recent studies have demonstrated that many vigorous newborns have temporary bradycardia immediately after birth [13,31] while a large proportion of newborns receiving resuscitation have HRs > 100 bpm [32,33]. As recently commented by Patterson and Niermeyer, [34] a dichotomous HR threshold may not suffice to determine the need for PPV. A more nuanced approach considering HR value, trajectory, and timing after birth may be more informative [32,33,34]. Video-recording evidence suggests that apnea or ineffective breathing more directly influences decision-making, and none of the ventilated newborns in the current study were breathing effectively at the commencement of PPV and thus all were in need of ventilation. The overall impression of the newborns’ conditions, including tone and color, may have been a contributing factor.

The user friendliness and safety of dry-electrode ECG were demonstrated in this trial, where the dry-electrode ECG device was placed in a real-world setting immediately after birth on 7000 newborns without the need for additional research personnel. The number of protocol violations where the HCPs removed the tape masking the HR display in compromised newborns reflects the HCPs’ perception of the importance of HR feedback during newborn resuscitation. ILCOR suggests that the potential advantages of rapid signal acquisition and continuous, accurate HR monitoring must be weighed against the potential costs of equipment and training [24]. The strengths of the dry-electrode ECG device include its affordability, minimal training requirements, and usability by a single provider, making it particularly advantageous in low-resource settings with the highest burden of newborns in need of resuscitation.

### Strengths and Limitations

The major strengths of this study were the population-based and randomized design, large study population, and recruitment of participants early in pregnancy, which limited the risk of selection bias. Another strength was that the indication for PPV was assessed from video recordings. The number of resuscitated newborns was in line with that reported previously from high-resource settings [17,35].

The main limitation was the early cessation of the trial halfway due to employee reservations about video-recording resuscitations. To prevent similar experiences in the future, we have established a multi-country, multi-center Safer Births Scandinavia collaboration for newborn resuscitation research. Secondly, the single-center design and low proportion of newborns receiving PPV implied a long trial period to reach the sample size. Randomization in uneven block sizes, unfortunately, resulted in an unintentional imbalance between groups, which should not affect the results of this study. Furthermore, the complex data collection with multiple data sources resulted in the loss of included participants. However, unstable video storage and inconsistent recording of time of birth and HR due to poor Bluetooth connection occurred randomly and should not affect the results. Ventilation monitoring data and HR at 60 s were only available for half of the ventilated newborns, and data should be interpreted with caution. Other limitations included manual registration of time of birth on a tablet, which is prone to human error, but a recent video study showed a high precision in documentation of time of birth at our hospital, with a median difference of 1.8 s between annotations and births captured on thermal cameras [36]. The structural constraint did not enable resuscitation with deferred cord clamping at our hospital, but this is in line with most settings globally, which adds to the generalizability. Both infrastructure and delay in decision-making regarding the need for respiratory support likely contributed to the non-adherence with resuscitation guidelines, in line with previous studies. The findings are generalizable for newborns born at ≥34 gestational weeks, who represent the majority of newborns that are resuscitated at birth. Lastly, the newborns that received PPV in the current study were mostly moderately asphyxiated, and the findings may not be generalizable to settings with higher rates of more severe birth asphyxia.

## 5. Conclusions

The use of dry-electrode ECG HR monitoring did not change the proportion of newborns that received PPV within 60 s after birth, but early termination due to employee protest over video recording rendered the trial inadequately powered to detect a difference. Newborns that received PPV were apneic or ineffectively breathing at the start of PPV, and only about a third were bradycardic. This reinforces current resuscitation guideline recommendations, highlighting that respiratory status is the most important indicator for starting PPV.

## Figures and Tables

**Table 1:** Maternal and newborn characteristics for the study participants receiving resuscitation with PPV by allocated group (n = 171).

Participant Characteristics	Intervention, n = 98	Control, n = 73	*p*-Value
Maternal age	31.0 (4.3)	31.8 (4.9)	0.2
Pre-eclampsia	3 (3.1%)	3 (4.1%)	0.7
Gestational diabetes	7 (7.1%)	4 (5.5%)	0.8
Parity			0.5
Nulliparous	56 (57%)	37 (51%)	
Primiparous	33 (34%)	26 (36%)	
Multiparous	9 (9.2%)	10 (14%)	
Mode of delivery			0.2
Vaginal	13 (13%)	18 (25%)	
Vacuum or forceps	32 (33%)	19 (26%)	
Elective caesarean section	2 (2.0%)	4 (5.5%)	
Emergency caesarean section	43 (44%)	28 (38%)	
Breech	8 (8.2%)	4 (5.5%)	
Gestational age (weeks)	39.2 (1.8)	39.3 (1.8)	0.8
Birth weight (grams)	3416 (736)	3540 (660)	0.4
Female	46 (47%)	32 (44%)	0.8
Twins	7 (7.1%)	4 (5.5%)	0.8
Umbilical arterial pH	7.16 (0.12)	7.19 (0.09)	0.3
Missing	18	14	
Apgar 1 min	5 (3, 6)	5 (4, 6)	>0.9
Apgar 5 min	8 (6, 9)	8 (7, 9)	0.4
Apgar 10 min	9 (8, 10)	9 (8,10)	0.6

Mean (SD); Median (25%, 75%); n (%); Wilcoxon rank sum test; Fisher’s exact test.

**Table 2:** Resuscitation characteristics and short-term clinical outcomes for resuscitated newborns (n = 171).

	Intervention, n = 98	Control, n = 73	*p*-Value
Resuscitation outcomes			
PPV provided within 60 s after birth	44 (45%)	24 (33%)	0.12
Time from birth to start of PPV (s)	66 (44, 101)	84 (50, 145)	0.058
Duration of PPV (s)	141 (66, 223)	122 (67, 231)	0.7
Missing	12	4	
HR data			
Heart rate at 60 s (bpm)	147 (88, 167)	139 (102, 168)	0.8
Missing	42	29	
Heart rate at start of PPV	146 (86, 177)	106 (75, 157)	0.065
Missing	49	29	
≥10 s of HR < 100 bpm in first 5 min after birth	37 (43%)	35 (60%)	0.06
Missing	12	15	
Tidal volumes			
Mean tidal volume first 30 s (mL/kg)	4.6 (3.1, 6.1)	4.8 (2.9, 7.9)	0.6
Mean tidal volume first 60 s (mL/kg)	4.7 (3.2, 6.4)	4.9 (2.8, 7.3)	0.6
Proportion of inflations with tidal volume ≥ 4 mL/kg first 30 s	0.6 (0.4, 0.8)	0.5 (0.2, 0.7)	0.12
Proportion of inflations with tidal volume ≥ 4 mL/kg first 60 s	0.6 (0.4, 0.8)	0.6 (0.3, 0.8)	0.7
Proportion of inflations with tidal volume ≥ 6 mL/kg first 30 s	0.3 (0.1, 0.5)	0.3 (0.0, 0.6)	0.3
Proportion of inflations with tidal volume ≥ 6 mL/kg first 60 s	0.3 (0.1, 0.5)	0.4 (0.1, 0.6)	0.8
Proportion of inflations with tidal volume ≥ 8 mL/kg first 30 s	0.1 (0.0, 0.3)	0.1 (0.0, 0.6)	0.6
Proportion of inflations with tidal volume ≥ 8 mL/kg first 60 s	0.2 (0.0, 0.3)	0.2 (0.0, 0.6)	0.5
Missing for all tidal volume data	46	35	
Short term outcomes			
Apgar at 5 min			0.4
Apgar ≥ 7	49 (50%)	41 (56%)	
Apgar < 7	49 (50%)	32 (44%)	
Apgar at 10 min			0.8
Apgar ≥ 7	80 (82%)	58 (79%)	
Apgar < 7	18 (18%)	15 (21%)	
Intubation	5 (5.1%)	1 (1.4%)	0.2
Chest compressions	4 (4.1%)	2 (2.7%)	>0.9
Death before discharge	1 (1.0%)	0 (0%)	>0.9

Median (25th, 75th quartiles); n (%); HR = heart rate; PPV = positive pressure ventilation.

## Data Availability

Data can be shared upon reasonable request.

## Abbreviations

The following abbreviations are used in this manuscript:

bpm	beats per minute
ECG	electrocardiogram
HCP	healthcare provider
HR	heart rate
ILCOR	The International Liaison Committee on Resuscitation
PPV	positive pressure ventilation
RCT	randomized controlled trial
